# Dual-function *Aspergillus sulphureoviridis*: antibacterial polyketides against *Dickeya solani* and elicitation of *Bacillus subtilis* lipopeptides

**DOI:** 10.1128/spectrum.00977-26

**Published:** 2026-07-20

**Authors:** Xinhui Wang, Carlos N. Lozano-Andrade, Scott A. Jarmusch, Jens C. Frisvad, Ákos T. Kovács, Thomas O. Larsen

**Affiliations:** 1 Collaborative Innovation Center for Modern Grain Circulation and Safety, and College of Food Science and Engineering, Nanjing University of Finance and Economics 12391, Nanjing, China; 2 DTU Bioengineering, Technical University of Denmark 5205, Kgs. Lyngby, Denmark; South China University of Technology, Guangzhou, China

**Keywords:** *Aspergillus sulphureoviridis*, molecular networking, antibacterial metabolites, *Dickeya solani*, microbial interactions, biocontrol

## Abstract

The Gram-negative bacterium *Dickeya solani* causes potato soft rot and blackleg, and effective control measures remain limited. We evaluated the agricultural potential of *Aspergillus sulphureoviridis*, a recently described and chemically unexplored fungus. Bioactivity-guided molecular networking of extracts that inhibited *D. solani* led to the isolation of six metabolites: cladobotrin IV (**1**), two new analogs cladobotrin VII and cladobotrin VIII (**2** and **3**), and the orsellinic acid-derived metabolites F9775A (**4**), F9775B (**5**), and violaceol I (**6**). Among these, the polyketide F9775B (**5**) inhibited both *Bacillus subtilis* and *D. solani* (IC_50_ 89.4 and 59.3 µg/mL, respectively), while cladobotrin VIII (**3**) was active against *B. subtilis* (IC_50_ 95 µg/mL). Furthermore, co-culture with the beneficial rhizobacterium *B. subtilis* revealed an additional interaction: *A. sulphureoviridis* induced a pronounced antifungal defense in *B. subtilis*. MALDI-MSI showed that this interaction spatially enriched canonical *Bacillus* lipopeptides (surfactins and fengycins) at the confrontation zone, whereas the shortened fengycins variants were detected only intracellularly within the *Bacillus* colony. This indicates that *A. sulphureoviridis* triggers *B. subtilis* to mobilize and secrete bioactive lipopeptides for defense while retaining the less active short-chain fengycin-like compounds intracellularly. Together, these results establish *A. sulphureoviridis* as both a source of antimicrobial compounds and an inducer of beneficial bacterial defenses, providing a rationale for further in-planta validation and development as a sustainable biocontrol agent.

**IMPORTANCE:**

Potato soft rot and blackleg caused by *Dickeya solani* are difficult to control, creating a need for sustainable biocontrol strategies. Here, we show that the recently described fungus *Aspergillus sulphureoviridis* has a dual function relevant to crop protection. Bioactivity-guided molecular networking enabled the targeted discovery of six antibacterial metabolites, including two new cladobotrin analogs and the first cladobotrins reported from *Aspergillus*, and identified F9775B as the main metabolite active against *D. solani*. Using MALDI mass spectrometry imaging, we further demonstrate that *A. sulphureoviridis* does not act only through its own metabolites but also triggers *Bacillus subtilis* to enrich and deploy surfactin and fengycins at the interaction zone. These findings expand the known chemical diversity of *A. sulphureoviridis* and support its potential as a dual-function contributor to sustainable microbe-based biocontrol.

## INTRODUCTION

Potato soft rot and blackleg, caused by bacterial pathogens within the genera *Dickeya* and *Pectobacterium*, rank among the most destructive plant diseases worldwide, leading to substantial economic losses in global potato production (1, 2). The management of these diseases, particularly those caused by the aggressive pathogen *Dickeya solani* (1), remains challenging due to the limited efficacy of chemical bactericides, escalating antimicrobial resistance, and growing environmental concerns. These challenges highlight the need for sustainable, mechanism-informed biocontrol strategies grounded in microbial natural products.

Within fungi, *Aspergillus* section Nidulantes is notable for rich secondary-metabolite capacity and biosynthetic gene-cluster (BGC) diversity, spanning both toxicological and pharmaceutically relevant scaffolds (3–6). *Aspergillus sulphureoviridis* is a recently described member of this section that has, until now, remained chemically unexplored, and its potential for agricultural applications is completely unknown (3). In our preliminary screen, crude extracts of *A. sulphureoviridis* inhibited the Gram-negative phytopathogen *D. solani* and the Gram-positive model *Bacillus subtilis*, which suggests broad antibacterial potential and motivated a targeted investigation of its secondary metabolome.

To efficiently identify the bioactive constituents responsible for this activity, we employed a bioactivity-based molecular networking strategy (7–9). This MS-guided approach enabled the prioritization and targeted isolation of antibacterial metabolites from the complex fungal extract (10). However, the ecological role and application potential of a microbial agent extend beyond the activity of its purified metabolites in a plate assay. In the rhizosphere, microbes exist in complex communities, and their interactions can profoundly influence the production of antimicrobial compounds. For instance, co-culture with fungi can trigger silent biosynthetic gene clusters in bacteria, leading to the production of novel defenses (11–13). We hypothesized that *A. sulphureoviridis* may not only directly antagonize plant pathogens through its own metabolites but also interact synergistically with beneficial rhizobacteria, potentially eliciting their biocontrol capacity.

To test this hypothesis and visualize the chemical interplay *in situ*, we extended our study to include a co-culture system combining *A. sulphureoviridis* with the beneficial bacterium *Bacillus subtilis*, a known producer of antifungal lipopeptides. We then applied matrix-assisted laser desorption/ionization mass spectrometry imaging (MALDI-MSI), a powerful technique that allows for the spatial mapping of metabolite production directly on the agar surface, revealing the molecular dynamics at the microbial interaction interface.

Accordingly, our aims were (i) to identify the key antibacterial metabolites from *A. sulphureoviridis* active against *D. solani* and (ii) to determine whether interaction with *B. subtilis* elicits a targeted antibacterial response, thereby evaluating *A. sulphureoviridis* as a potential dual-function contributor to sustainable crop protection.

## MATERIALS AND METHODS

### General experimental procedures

The NMR spectra were recorded on a Bruker AVANCE III 800 MHz spectrometer (Bruker, 279 Billerica, MA, USA) equipped with a 5 mm TCI CryoProbe using standard pulse sequences. The ^1^H and ^13^C NMR chemical shifts were reported with reference to the residual solvent signals at *δ*_H_ 7.26 and *δ*_C_ 77.16 ppm for CDCl_3_, *δ*_H_ 3.31 and *δ*_C_ 49.00 ppm for CD_3_OD, *δ*_H_ 2.50 and *δ*_C_ 39.52 ppm for (CD_3_)_2_SO. UHPLC-HRMS was performed on an Agilent Infinity 1290 UHPLC (Agilent Technologies, Santa Clara, CA, USA) system equipped with a diode array detector. UV-vis spectra were recorded from 190 to 640 nm. All solvents and chemicals used for HRMS and chromatography were LC-MS grade, while the solvents for metabolites extraction were of HPLC grade. Water was purified using a Milli-Q system (Millipore, Bedford, MA, USA).

### Fungal strains

*A. sulphureoviridis* IBT 21868 was obtained from the IBT culture collection, Department of Biotechnology and Biomedicine, Technical University of Denmark.

### Small-scale cultivation and extraction

The fungus was three-point inoculated on 35 YES plates and incubated for 14 days in the dark at 25°C. Extraction was performed twice with acidic EtOAc (1% formic acid).

### Large-scale cultivation, extraction, and isolation

The fungus was three-point inoculated on 200 YES plates and incubated for 14 days in the dark at 25°C, whereafter they were extracted twice with acidic EtOAc (1% formic acid).

The dried extract (9 g) was dissolved in 1:9 Milli-Q water-MeOH and extracted with heptane. After separating two phases, enough Milli-Q water was added to the water-MeOH phase to reach a ratio of 1:1; followed by extraction with dichloromethane (DCM), yielding three phases in total (Milli-Q water-MeOH, DCM, and heptane). The crude extract from the DCM phase was dried and fractionated on a 25 g RP C_18_ (Grace, 15 µm/100 Å) flash column (Biotage, Uppsala, Sweden) using the Isolera One automated flash system (Biotage). The gradient used was 10%–100% MeOH over 35 min (25 mL/min). Fractions were automatically collected based on UV signal (230 nm and 280 nm). The 60% subfraction was further fractionated using a linear gradient from 25 to 42 MeCN in Milli-Q water over 30 min by an Agilent Infinity 1290 HPLC-DAD (Agilent Technologies, Santa Clara, CA, USA) system, with a flow rate of 4 mL/min, UV monitoring at 230, 280, and 330 nm and column temperature at 40°C to get 14 subfractions (S1–S14). Cladobotrin IV (**1**, 3.6 mg) and Cladobotrin VII (**2**, 0.5 mg) were obtained from S2 on a Gemini C_6_-Phenyl column (5 μm, 110 Å, 250 × 10 mm, Phenomenex) using a linear gradient from 20% to 30% MeCN in Milli-Q water over 30 min; Cladobotrin VIII (**3**, 0.4 mg) was purified from S2 on a RP C_18_ column (5 μm, 100 Å, 250 × 10 mm, Phenomenex) using a linear gradient from 15% to 20% MeCN in Milli-Q water over 30 min to yield; F9775A (**4**) was purified from S9 on a Gemini C_6_-Phenyl column (5 μm, 110 Å, 250 × 10 mm, Phenomenex) using a linear gradient from 29% to 37% MeCN in Milli-Q water over 30 min; F9775B (**5**) was purified from S5 on a Gemini C_6_-Phenyl column (5 μm, 110 Å, 250 × 10 mm, Phenomenex) using a linear gradient from 27% to 34% MeCN in Milli-Q water over 30 min; violaceol I (**6**) was purified from 70% subfraction on a RP C_18_ column (5 μm, 100 Å, 250 × 10 mm, Phenomenex) using a linear gradient from 25% to 37% MeCN in Milli-Q water over 30 min to yield.

### Data-dependent LC-ESI-HRMS^2^ analysis

UHPLC-DAD-QTOFMS was performed on an Agilent Infinity 1290 UHPLC system equipped with a diode array detector. Separation was achieved on a 250 × 2.1 mm i.d., 2.7 µm, Poroshell 120 Phenyl-Hexyl column (Agilent Technologies, Santa Clara, CA) held at 60°C. Mass spectrometry (MS) detection was performed as previously described (14). All samples were prepared at a concentration of 5 mg/mL and injected 1 µL. In-house fungal metabolite library search was done as described by Kildgaard et al. (15).

### Bioactivity-based molecular networking

The MS/MS data file was converted from Agilent MassHunter data files (.d) to the mzXML file format with MS-Convert and subjected to MZmine 2.53 analysis. The mass detection was performed using a centroid algorithm with a noise level of 4,000 for the MS1 level and 50 for MS2 level. The ADAP chromatogram builder was established using a minimum group size of scans of 5, a group intensity threshold of 4,000, a minimum highest intensity of 2e4, and an *m/z* tolerance of 0.01 Da. The peak deconvolution was performed using an ADAP wavelets algorithm with the following standard settings: *S*/*N* threshold = 10, minimum feature height = 3E4, coefficient/area threshold = 40, peak duration range 0.02–1.0 min, RT wavelet range 0.01–0.05. MS/MS scans were paired using an *m/z* tolerance range of 0.02 Da and RT tolerance range of 1.0 min. The chromatograms were deisotoped with *m/z* tolerance of 0.01 and retention time tolerance of 0.1 min. The duplicate peaks were filtered with *m/z* tolerance of 0.016 and retention time tolerance of 0.1 min. The peak list was aligned using the join aligner algorithm with *m/z* tolerance of 0.001, weight for *m/z* = 2, weight for RT = 0.5, and retention time tolerance of 0.3 min. Eventually, the mgf data file with MS/MS spectra and its corresponding .csv file were exported. The spectral information (.mgf) data file was uploaded to the GNPS Web platform (http://gnps.ucsd.edu) for generating MS/MS molecular network with MS-Cluster deactivated. The data were filtered by removing all MS/MS fragment ions within ±17 Da of the precursor *m/z*. MS/MS spectra were window filtered by choosing only the top six fragment ions in the ±50 Da window throughout the spectrum. The precursor ion mass tolerance was set to 0.1 Da and the MS/MS fragment ion tolerance to 0.05 Da. A molecular network was then created where edges were filtered to have a cosine score above 0.65 and more than 4 matched peaks. The molecular networks were visualized using Cytoscape software (16).

An R-based Jupyter notebook, called Bioactive Molecular Networks available at GitHub, https://github.com/DorresteinLaboratory/Bioactive_Molecular_Networks, was used to determine the bioactivity score for each ion in the samples. The Pearson correlation score (*r*) and its significance between the peak area of an ion and the bioactivity level were obtained after the samples were normalized by normalizing the intensity of the TIC. The outputted node attribute table was incorporated into the Cytoscape software to visualize the molecular network and to map out the bioactivity scores.

### Sample preparation for mass spectrometry imaging

The co-culture experiments were performed in 9 cm Petri dishes containing 10 mL CYA by plating 5 μL of each microbial suspension. The *A. sulphureoviridis* was inoculated first at 25°C, and *B. subtilis* was inoculated 2 days later at 2.5 cm from the fungal colony. Then, both microbials were inoculated for 3 days more together. After incubation on thin agar plates, the agar layer was cut from the plates and used for MALDI imaging. DHB matrix solution was prepared in acetonitrile−MeOH−H_2_O (70/25/5 vol:vol:vol) at 20 mg/mL was deposited evenly over the glass (Bruker, IntelliSlides) with a sprayer (HTX Technologies), and the agar layer was placed on top of the slide, followed by overnight drying in a desiccator. A second layer of matrix was then sprayed onto the agar surface. The sample was inserted into a Bruker Autoflex Speed MALDI-TOF for data collection.

### Antibacterial assay

The fractions were tested for antibacterial activity against *B. subtilis* 3610 and *D. solani* using a diffusion plate assay. Briefly, overnight cultures of both strains were diluted (1:100) on warm LB agar and poured into petri dishes containing a gelled layer of LB agar (1.5%). After 10 min of drying, 6-mm wells were punched in the agar plates, and 80 µL of each extract was dispensed into the well. A kanamycin (5 µg/mL) solution was used as a positive control. The plates were incubated for 24 h at 30°C. All the experiments were conducted with three biological replicates.

The pure compounds for antibacterial activity test against *B. subtilis* 3610 and *D. solani* were dissolved in DMSO to a stock solution of 10 mg/mL and were serially diluted in DMSO with a dilution factor of 2 to provide 10 concentrations starting at 96 µg/mL for all the antibacterial assays. Kanamycin was included as a positive control for the purified-compound antibacterial assay and was tested under the same assay conditions using a twofold serial dilution over a concentration range of 0.125–64 µg/mL. The experimental details were followed previously described methodology (17, 18).

## RESULTS

### Discovery of Broad-spectrum antibacterial activity and a rich metabolome in *Aspergillus sulphureoviridis*

To explore sustainable solutions against bacterial phytopathogens, a panel of fungal isolates was screened for antibacterial activity. Crude extracts of *A. sulphureoviridis*, a previously uncharacterized species, exhibited strong inhibition against both a Gram-negative plant pathogen (*D. solani*) and a Gram-positive model bacterium (*B. subtilis*), producing clear zones of inhibition (Fig. S1 and S2). This broad-spectrum activity identified *A. sulphureoviridis* as a promising source of novel anti-phytobacterial metabolites.

To elucidate the chemical basis of this bioactivity, comprehensive metabolomic profiling was performed for *A. sulphureoviridis* cultivated on Czapek yeast agar (CYA) and yeast extract sucrose agar (YES). UHPLC–DAD–QTOF-MS analysis coupled with dereplication against multiple databases revealed a chemically diverse secondary metabolome comprising ophiobolins, nidulanins, pseurotin A, and emericellamides (Table 1). This remarkable chemical diversity underscores significant biosynthetic capacity of *A. sulphureoviridis* and provides a roadmap for targeted isolation of novel compounds with potential agricultural relevance.

**TABLE 1:** Summary of the dereplicated compounds in *A. sulphureoviridis*

No.	Names and references	Formula	RT	Adduction	*m*/*z*	Mass (MFG)^*a*^	Error (ppm)
1	Ophiobolin-B (19)	C_25_H_38_O_4_	8.134	[M + H]^+^	403.2844	402.2770	−0.28
2	1,2-Dilinoleoyl-sn-glycero-3-phosphocholine (20)	C_44_H_80_NO_8_P	10.703	[M + H]^+^	782.5699	781.5622	−0.60
3	Violaceol I (21, 22)	C_14_H_14_O_5_	4.659	[M + H]^+^	263.0912	262.0841	0.76
4	Pseurotin A (23)	C_22_H_25_NO_8_	4.707	[M + Na]^+^	454.1446	431.1553	0.09
5	Ergosterol (24)	C_28_H_44_O	10.389	[M + H]^+^	397.3458	396.3392	1.75
6	Ophiobolin H (19)	C_25_H_38_O_3_	8.63	[M + H]^+^	387.2898	386.2821	−1.11
7	Nidulanin A (25)	C_34_H_45_N_5_O_5_	8.194	[M + H]^+^	604.3498	603.3421	−0.75
8	4-Hydroxy-3,6-dimethyl-2H-pyran-2-one (26)	C_7_H_8_O_3_	2.502	[M + H]^+^	141.0544	140.0473	1.57
9	Phytosphingosine (27)	C_18_H_39_NO_3_	6.461	[M + H]^+^	318.3001	317.2930	0.54
10	Nidulanin D-II (28)	C_29_H_37_N_5_O_5_	8.367	[M + H]^+^	536.2866	535.2795	0.27
11	Nidulanin D-I	C_29_H_37_N_5_O_5_	6.871	[M + H]^+^	536.2868	535.2795	−0.1
12	Nidulanin B-II	C_34_H_45_N_5_O_6_	7.468	[M + H]^+^	620.3442	619.3370	0.1
13	Ergokonin B (29)	C_28_H_42_O_5_	9.201	[M + Na]^+^	481.2916	458.3032	1.84
14	Emericellamide H (30)	C_33_H_59_N_5_O_7_	7.751	[M + H]^+^	638.4493	637.4414	−0.9
15	Emericellamide G	C_29_H_51_N_5_O_7_	6.597	[M + H]^+^	582.3858	581.3788	0.56
16	Emericellamide E	C_32_H_57_N_5_O_7_	7.575	[M + H]^+^	624.4337	623.4258	−1.0
17	Emericellamide C	C_30_H_53_N_5_O_7_	6.806	[M + H]^+^	596.4022	595.3945	−0.71
18	Emericellamide B (31)	C_34_H_61_N_5_O_7_	7.942	[M + Na]^+^	674.4466	651.4571	−0.43
19	Emericellamide A	C_31_H_55_N_5_O_7_	7.35	[M + H]^+^	610.4179	609.4101	−0.78
20	Cyclo-(Phe-Val-Val-Phe)	C_28_H_36_N_4_O_4_	8.9	[M + H]^+^	493.2810	492.2737	−0.14

^*a*^ This column shows that the results were found by the Generate Formulas algorithm (MGF) in MassHunter.

### Bioactivity-based molecular networking reveals candidate antibacterial metabolites

Next, for bioactivity-guided discovery, *A. sulphureoviridis* was cultivated on 35 YES agar plates, and the combined culture extracts were fractionated using reversed-phase flash chromatography on a C18 column (10 g) with a linear gradient from 10% to 100% MeOH in water, yielding 12 fractions. At a concentration of 5 mg/mL, fractions 5–8 exhibited the strongest antibacterial activity (>50% inhibition) against *B. subtilis* and *D. solani* (Fig. S1 and S2). Each fraction was analyzed by UHPLC–DAD–TOF-MS to generate feature tables for molecular networking.

To correlate chemical features with bioactivity, a bioactivity-based molecular networking analysis was performed. After the data files were pre-processed by MZmine, the exported file was uploaded to the GNPS Web platform (8). Next, the bioactivity score was defined as the Pearson correlation coefficients (*r*), which were calculated between the relative abundances of the molecular ions (peak area) in the fractions and their antibacterial activities (present as inhibition rate here) (10). The activity results against both pathogenic bacteria were used to calculate bioactivity scores and generate bioactive molecular networks respectively, followed by visualization using Cytoscape (16). Nodes with positive bioactivity scores approaching one were considered likely bioactive metabolites.

**Fig 1:**
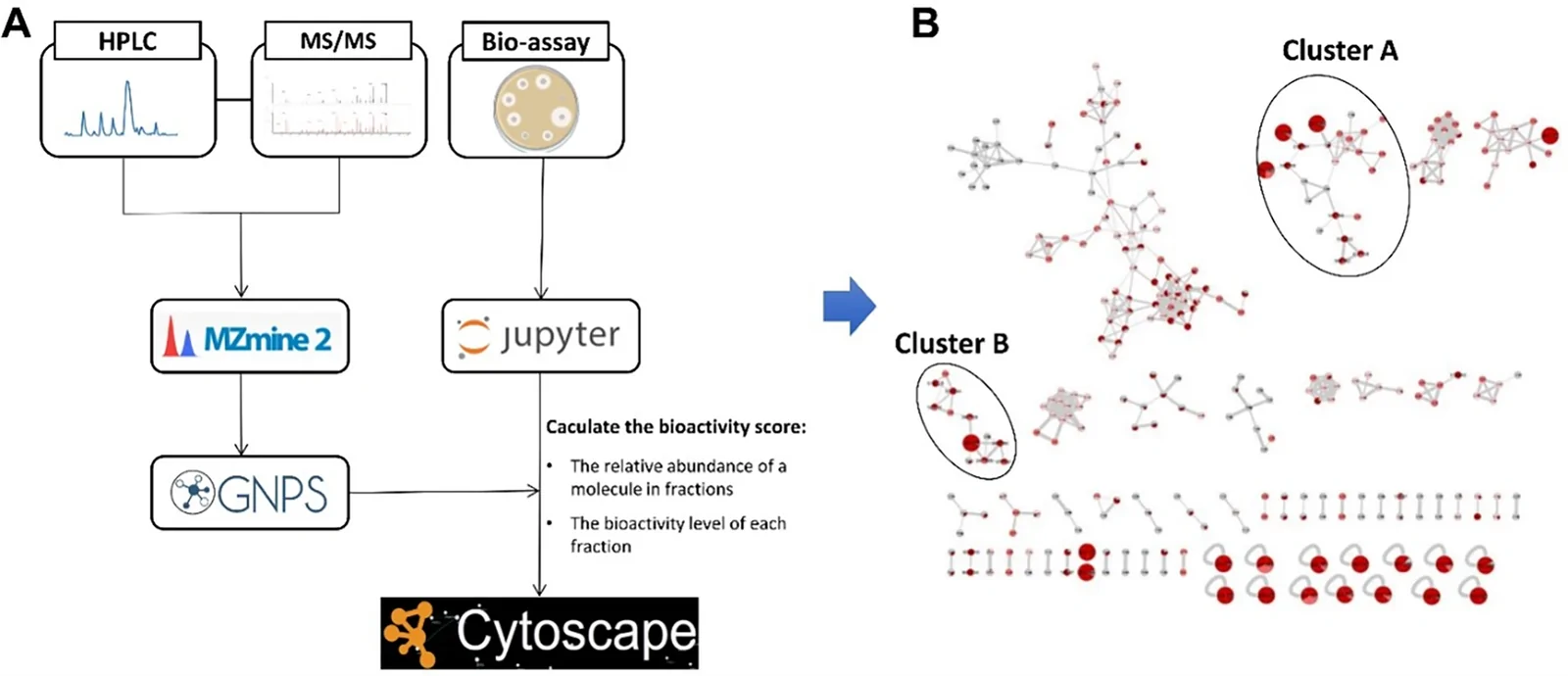
Workflow of bioactivity-guided molecular networking and identification of active metabolites. (**A**) Schematic workflow integrating HPLC fractionation, MS/MS analysis, and bioassay results (10). (**B**) The full bioactive molecular network of the fractionated *A. sulphureoviridis* extract against *Dickeya solani*, highlighting two main clusters (**A and B**).

The resulting networks contained 868 nodes forming several clusters (Fig. 1 and Fig. S3 and S4). For both *B. subtilis* and *D. solani*, the highest bioactivity scores (*r* > 0.65, *P* < 0.02) occurred for multiple nodes in clusters A and B. As the two networks exhibited highly similar patterns, only the *D. solani* network is presented in the main text. Therefore, compounds in these clusters showing both strong bioactivity and relatively high abundance were prioritized for purification, as they were most likely responsible for the observed inhibition of *B. subtilis* and *D. solani*.

### Bioactivity-guided isolation identifies F9775B as a potent anti-phytobacterial metabolite

**Fig 2:**
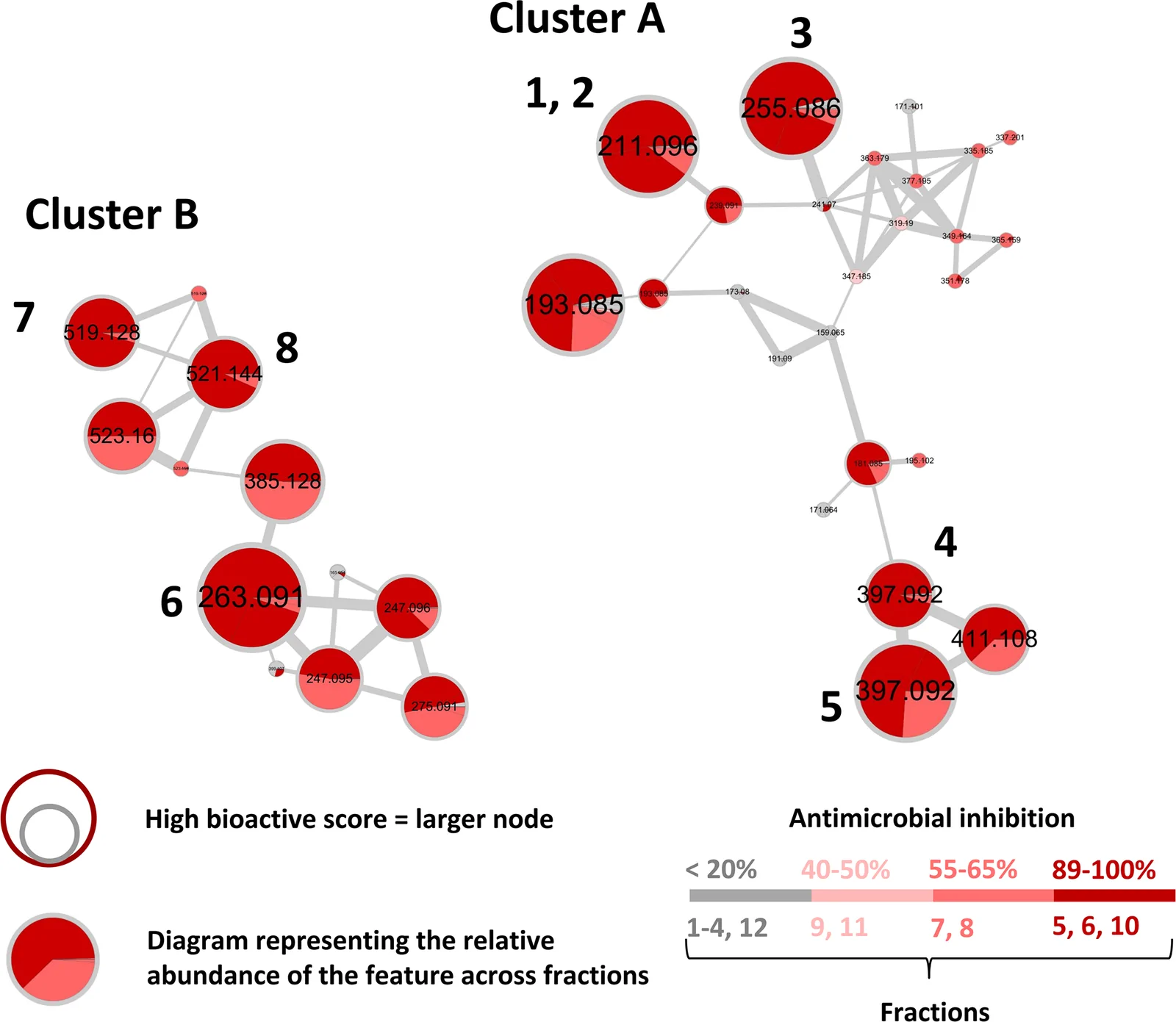
Bioactive molecular network of the fractionated *A. sulphureoviridis* extract against *Dickeya solani* (Bioactive molecular network results against *Bacillus subtilis* are shown in Fig. S4).

Based on both bioactivity and the potential for structural novelty, compounds **1**–**6** were selected for isolation from clusters A and B. One node in cluster A (*m/z* 211.0970) was dereplicated as cladobotrin IV (**1**), (32) which was identified for the first time from this genus (Fig. S5 through S8). No other nodes in clusters A or B matched entries in the GNPS library or in-house DTU database (Fig. 2). Large-scale cultivation of *A. sulphureoviridis* on YES agar, followed by targeted isolation guided by molecular network analysis, yielded two new compounds (**2** and **3**) and four known metabolites: cladobotrin IV (**1**), F9775A (**4**), F9775B (**5**), and violaceol I (**6**).

**Fig 3:**
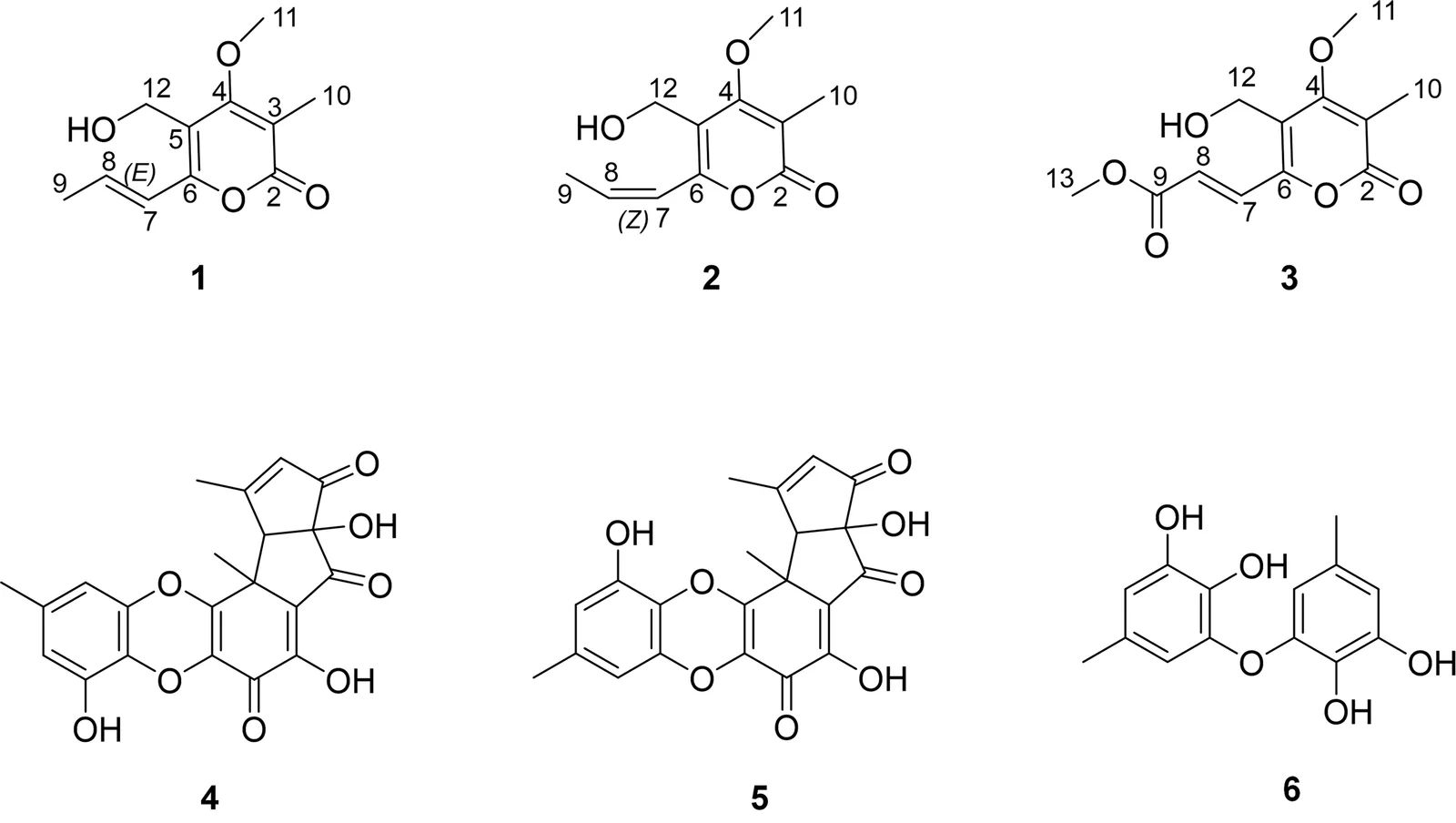
Structures of isolated compounds.

**Fig 4:**
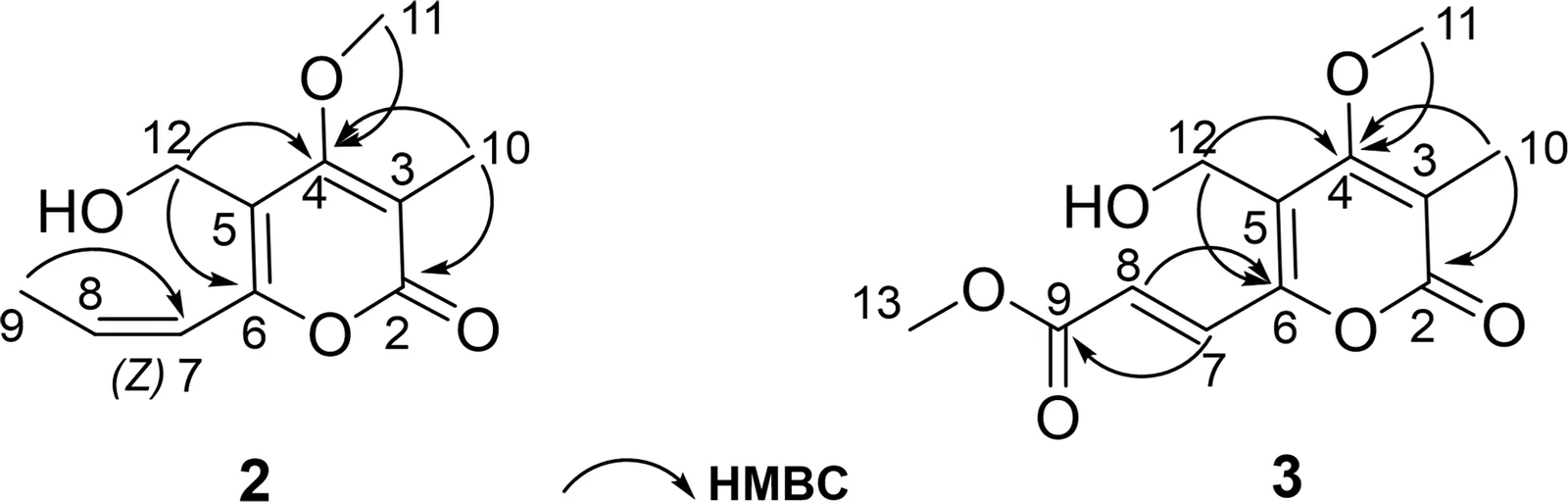
Key HMBC correlations of new compounds 2 and 3.

Compound **2**, which was obtained as a colorless crystal, was assigned the molecular formula C_11_H_14_O_4_ based on the ESI HRMS peak at *m/z* 211.0970 [M + H]^+^ (calcd. 211.0965), suggesting a molecule with five double bond equivalents (DBE), exactly as also seen for **1**. The UV spectrum showed absorption maxima at 235 and 325 nm further indicating that **2** was a structural isomer of **1**. In line with this, the ^1^H NMR spectrum of **2** revealed the presence of two olefinic protons, an oxygenated methylene, a methoxy group, and two methyl groups. Detailed analysis of the ^1^H, ^13^C, and HSQC NMR spectroscopic data of **2** confirmed the presence of 11 carbons, including 1 carbonyl group, 4 non-protonated sp^2^ carbons, 2 olefinic methine carbons, and 2 methyl carbons. The HMBC correlations of H2-12 to C-4, C-5, and C-6, H3-11 to C-4, and H3-10 to C-2, C-3, and C-4 suggested the presence of an α-pyrone core structure (Fig. S9 through S11). HMBC correlations from H-7 to C-6, C-8, and C-9 and from H-8 to C-6, C-7, and C-9 established the connection of the α-pyrone substructure to the propenyl chain (Table 2). Compounds **2** and cladobotrin IV (**1**) have almost the same fragmentation patterns but elute at different retention times. However, in the ^1^H spectrum, the coupling constant of olefinic protons in **2** (^3^*J* = 11.82 Hz) is smaller than **1** (^3^*J* = 15.22 Hz), which indicated it to be the *cis*-isomer of **1** (33). Based on these data, the gross structure of cladobotrin VII (**2**) was established as shown in Fig. 3 and 4.

**TABLE 2:** ^1^H (800 MHz) and ^13^C NMR (200 MHz) data for cladobotrin IV, VII, and VIII (**1**–**3**)

No.	1^*b*^			2^*c*^			3^*b*^		
	*δ*_H_, (*J* in Hz)	*δ_C_*	HMBC	*δ*_H_, (*J* in Hz)	*δ*_C_	HMBC	*δ*_H_, (*J* in Hz)	*δ*_C_	HMBC
2		165.08			165.6			163.4	
3		110.35			109.6			114.4	
4		167.98			168.78			166.51	
5		112.0			114.8			118.6	
6		155			156.92			151.82	
7	6.38 (15.22, 1.73)	119.45	6, 8, 9	6.39 (11.82, 1.72)	119.01	^*a*^	7.56	130.5	6, 8, 9
8	6.76 (7.0, 15.22)	136	6, 7, 9	6.13 (7.5, 11.82)	136.94		6.77	124.37	6, 7, 9
9	1.92 (7.0, 1.55)	18.84	7, 8	2.10 (7.5, 1.72)	15.82	7, 8		166.52	
10	2.05	10.66	2, 3, 4	2.04	10.21	2, 3, 4	2.13	11.08	2, 3, 4
11	3.91	61.45	4	3.95	61.87	4	3.97	61.59	4
12	4.49	55.37	4, 5, 6	4.44	54.7	4, 5, 6	4.60	55.13	4, 5, 6
13	−^*d*^	−	−	−	−	−	3.81	52.2	8, 9

^*a*^ The signals are too weak to be recorded.

^*b*^ Compounds 1 and 3 were measured in CDCl3.

^*c*^ Compound 2 was measured in CD3OD.

^*d*^ "–" indicates not applicable (compounds 1 and 2 do not contain the corresponding C-13 position).

Compound **3**, obtained as a colorless amorphous solid, was assigned the molecular formula C_12_H_14_O_6_ based on the ESI HRMS peak at *m/z* 255.0869 [M + H]^+^ (calcd. 255.0863), suggesting the double bond equivalent (DBE) of six. The UV spectrum showed absorption maxima at 242 and 340 nm. The ^1^H NMR spectrum of **3** revealed the presence of two olefinic protons, an oxygenated methylene, two methoxy groups, and one methyl group. Detailed analysis of the ^1^H, ^13^C, and HSQC NMR spectroscopic data of **3** revealed the presence of 12 carbons, including 2 carbonyl groups, 4 non-protonated sp^2^ carbons, 2 olefinic methine carbons, and 1 methyl carbon. The HMBC correlations from H_3_-10 to C-2, C-3, and C-4, H_2_-12 to C-4, C-5, and C-6, and H_3_-11 to C-4, suggested the presence of the α-pyrone core structure like that seen in compounds **1** and **2** (Fig. 4 and Fig. S12 through S15). In addition, HMBC correlations from H-7 to C-6, C-8, and C-9, from H-8 to C-6, C-7, and C-9 and from H_3_-13 to C-8 and C-9 showed the presence of a methyl acrylate moiety at C-6 of the pyrone (Table 2). The planar structure of **3** as shown in Fig. 3 was established. These results confirmed that cladobotrin VIII (**3**) is a new derivative of **1**.

The identities of known compounds cladobotrin IV (**1**), F9775A (**4**), F9775B (**5**), and violaceol I (**6**) were confirmed by comparison with literature NMR data (Fig. 3) (21, 22, 32, 34).

Moreover, two additional low-abundance metabolites *m/z* 519.1286 (**7**) and *m/z* 521.1448 (**8**) were detected and annotated as novel features. Their molecular weights and MS/MS fragmentation patterns were established although full structural elucidation was not possible due to insufficient quantities (Table 3). Molecular networking analysis indicated that these metabolites are closely associated with violaceol I (**6**), suggesting a potential biosynthetic relationship.

**TABLE 3:** Information of potential bioactive compounds

*m/z* [M + H]^+^	Calc. [M + H]^+^	Molecular formula	RT (min)	MS/MS fragments
519.1286 (**7**)	519.1286	C_28_H_22_O_10_	5.27	501.1186; 380.0898; 153.0547
521.1448 (**8**)	521.1442	C_28_H_24_O_10_	5.53	503.1331; 382.1051; 137.0596

Next, we evaluated the antimicrobial activities of purified compounds **1**–**6**. The newly discovered cladobotrin VII (**2**) and cladobotrin VIII (**3**) showed only weak activity against the target pathogens (Table 4). In contrast, the polyketide F9775B (**5**) emerged as a moderate broad-spectrum antibacterial agent. It exhibited inhibitory activity against *B. subtilis* (IC_50_ = 89.4 µg/mL) and, more importantly, against the plant pathogen *D. solani* (IC_50_ = 59.3 µg/mL). Cladobotrin VIII (**3**) showed selective inhibition of *B. subtilis* (IC_50_ = 95 µg/mL) but showed no activity against *D. solani*, and the other compounds (**1**, **4**, **6**) had IC_50_ > 100 µg/mL for both bacteria (Table 4). Thus, F9775B (**5**) was identified as the primary anti-*D. solani* metabolite responsible for the observed growth inhibition.

**TABLE 4:** Antimicrobial activity of compounds **1**–**6**

Compound	*B. subtilis*		*D. solani*	
	Inhibition rate (%, 100 µg/mL)	IC_50_ (µg/mL)	Inhibition rate (%, 100 µg/mL)	IC_50_ (µg/mL)
**1**	−	>100	−^*a*^	>100
**2**	47.9	>100	25.9	>100
**3**	66.5	95	−	>100
**4**	37.1	>100	−	>100
**5**	61.4	89.4	57.7	59.3
**6**	36.3	>100	−	>100

^*a*^ "–" indicates no inhibition was observed at 100 µg/mL.

### Co-culture with *A. sulphureoviridis* elicits and reshapes the lipopeptide profile of *B. subtilis*

**Fig 5:**
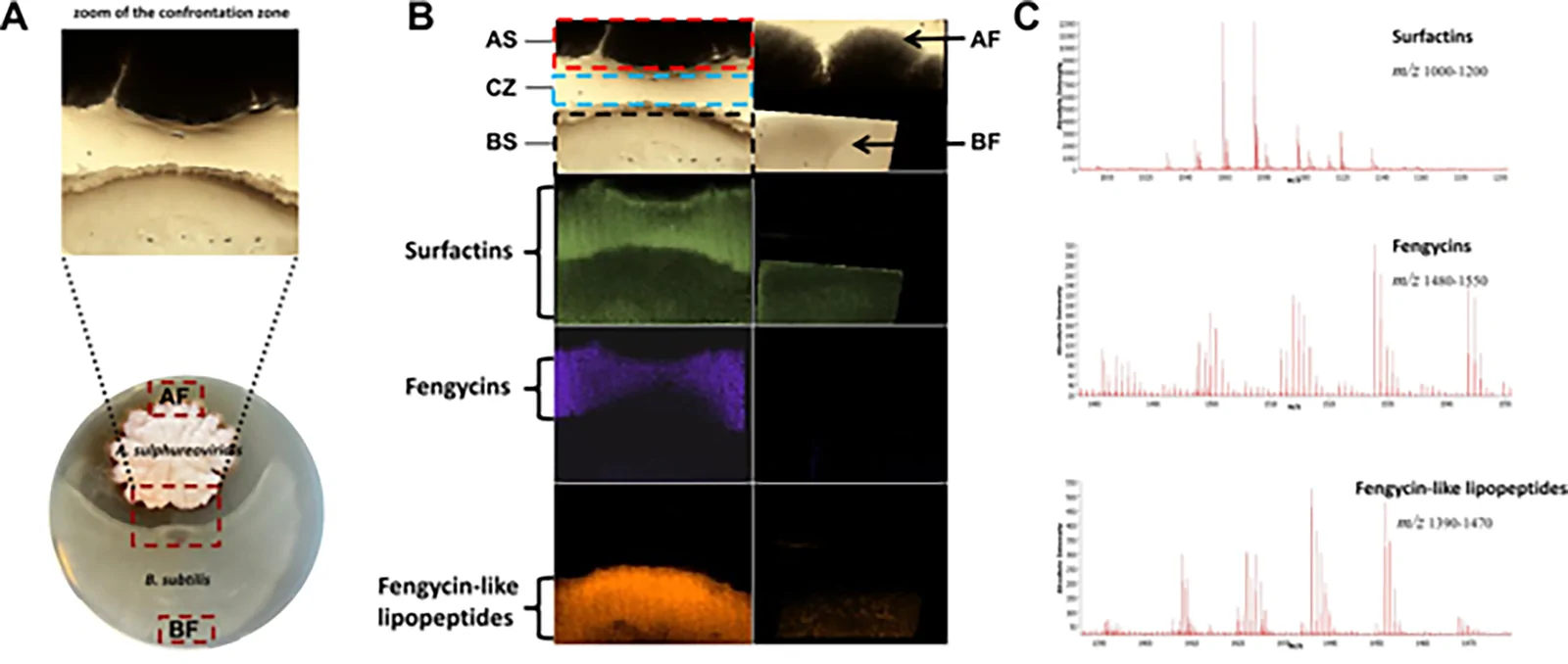
The spatial distribution of the cyclic lipopeptides deployed against *A. sulphureoviridis*. (**A**) Interaction between *B. subtilis* (lower colony) and *A. sulphureoviridis* (upper colony) on a CYA plate. The red dashed rectangle marks the region selected for MALDI imaging. Zoom of the confrontation zone of the MALDI scanning image is shown above. (**B**) MALDI–MSI visualization of metabolite distribution across five regions: the *A. sulphureoviridis* region (AS), the confrontation zone (CZ), the *B. subtilis* region (BS), the *A. sulphureoviridis* colony front (AF), and the *B. subtilis* colony front (BF). The ion images reveal the spatial distributions of three major groups of Bacillus-derived lipopeptides: surfactins, fengycins, and fengycin-like lipopeptides. Signal intensities are presented as false-color heat maps. (**C**) Representative mass spectra for the three lipopeptide families detected by MALDI-MSI. Surfactins (*m/z* 1,030.6–1,140.5), fengycins (*m/z* 1,469.8–1,527.8), and fengycin-like lipopeptides (*m/z* 1,391.8–1,451.8) exhibit distinct ion clusters corresponding to different structural variants.

Given that *Bacillus subtilis* is a common and beneficial bacterium in the soil ecosystem, we co-cultured *A. sulphureoviridis* with this rhizobacterium to explore potential synergistic interactions in the natural state. Metabolites were then mapped *in situ* using MALDI-MSI. A clear zone of inhibition formed at the confrontation zone (Fig. 5A and Fig. S16), indicating bilateral antagonism. Ion images were generated for five spatial zones (Fig. 5B): the *B. subtilis* region farthest (BF) and nearest (BS) to the fungus, the *A. sulphureoviridis* region farthest (AF) and nearest (AS) to the bacterium, and the confrontation zone (CZ) in between.

MALDI–MSI revealed a spatially resolved distribution of *B. subtilis* lipopeptides. Surfactins were enriched at the CZ and extended onto the fungal surface (Fig. 5B and C and Table 5) (35). Fengycin homologs were detected almost exclusively at the CZ, indicating co-culture induced accumulation (Fig. 5B and C and Table 5).

**TABLE 5:** List of putative assignments of sodium adducts of surfactins, fengycins, and fengycin-like lipopeptides during competition by positive MALDI-TOF imaging (35).

[M + Na]^+^	[M + K]^+^	Assignments
1,391.84	1,407.83	Fengycin-like lipopeptides
1,405.86	1,421.84	Fengycin-like lipopeptides
1,419.88	1,435.84	Fengycin-like lipopeptides
1,433.83	1,449.80	Fengycin-like lipopeptides
1,451.83	1,467.78	Fengycin-like lipopeptides/C14 fengycin A derivative
1,469.78	1,485.76	Monounsaturated C13 fengycin A
1,483.8	1,499.78	Monounsaturated C14 fengycin A
1,485.78	1,501.76	C14 fengycin A
1,497.82	1,513.8	Monounsaturated C15 fengycin B
1,499.8	1,515.78	C15 fengycin A
1,511.83	1,527.81	Monounsaturated C16 fengycin B
1,513.81	1,529.79	C16 fengycin B
1,527.83	1,543.81	C17 fengycin B
1,030.61	1,046.58	C13 surfactin
1,044.61	1,060.59	C14 surfactin
1,058.66	1,074.60	C15 surfactin
1,096.48	1,112.57	Surfactin derivative
1,102.61	1,118.57	Surfactin derivative
1,134.54	1,150.50	Surfactin derivative
1,140.54	1,156.53	Surfactin derivative

**Fig 6:**
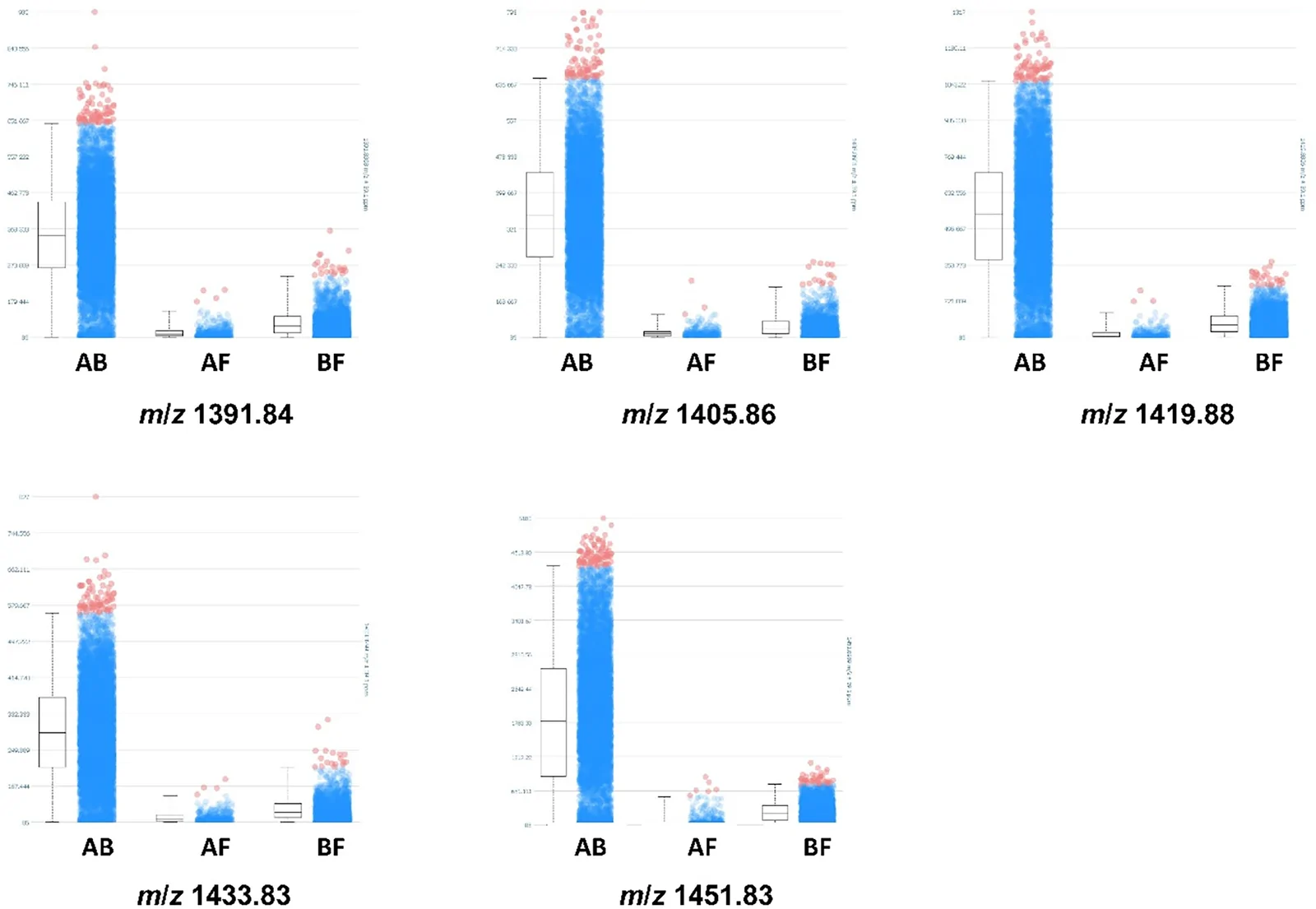
Intensity box plot comparisons of unidentified lipopeptides for [M + Na]^+^ from SCiLS software. Each dot represents the normalized peak intensity of a unique mass spectrum, corresponding to a single annotation on the culture. The center line in the box indicates the median, and the lower and upper boundaries of the box indicate the second and third quartiles. The vertical lines protruding from the box designate the lower (0%) and upper quantile (99%).

Notably, additional lipopeptide ions (*m/z* 1,391.8–1,451.8) appeared only within the *Bacillus* colony and had increased production in the BS region compared to the BF zone (Fig. 5B and C and Table 5). AntiSMASH analysis of the *B. subtilis* genome revealed only the canonical fengycin and surfactin NRPS clusters (Fig. S17), excluding *de novo* biosynthesis and supporting that these ions represent fengycins-related derivatives. Consistent with the MSI data, the intensity distributions of this fengycins-like derivatives also showed strong enrichment in the BS but were nearly absent at the colony edges (BF) (Fig. 6).

Compared with the main groups of fengycin ions, these fengycins-like derivatives (*m/z* 1, 391.8–1,451.8) exhibited slightly lower molecular masses. The small mass differences make amino-acid truncation within the peptide backbone unlikely. Instead, the shifts are more plausibly attributed to alterations in the lipid moiety, such as ring opening, partial fatty-acid chain shortening, or dehydration-related modifications. Because the current MALDI-MSI data set lacks sufficient mass resolution to deduce accurate molecular formulas, the exact neutral losses and structural alterations cannot yet be defined. Future work will incorporate targeted MS/MS analyses and isolation of these modified congeners to determine whether their fragmentation behavior and core structures correspond to the canonical fengycin scaffold.

**Fig 7:**
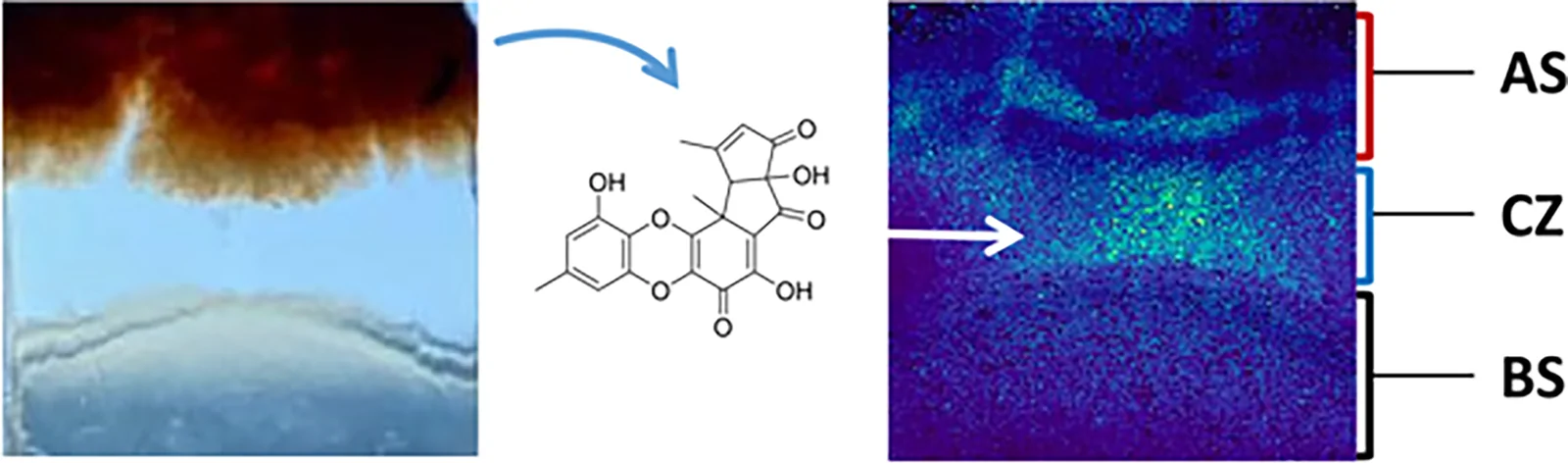
Spatial localization of the antibacterial metabolite F9775B from fungus at the confrontation zone. The left shows the optical image of the interaction interface between *A. sulphureoviridis* and *B. subtilis*. The right presents the MALDI–MSI ion image displaying the spatial distribution of F9775B (**5**) at the microbial interface. The detection of **5** in the confrontation zone (CZ) and on the surface of the *B. subtilis* colony indicates that the fungus exhibits an antagonistic chemical response in the presence of *B. subtilis*.

Consistent with the bioactivity-guided isolation results, the fungal polyketide F9775B (**5**) was detected at the confrontation zone (CZ) in the co-culture system (Fig. 7). In regions distant from the interaction interface, F9775B was mainly confined within the fungal mycelium, whereas at the CZ, it extended beyond the mycelial boundary and accumulated at the interaction front. This spatial localization provides ecological context for the *in vitro* antibacterial activity of F9775B against *D. solani*.

## DISCUSSION

The difficulty of controlling aggressive phytopathogens such as *D. solani*, together with limitations of conventional pesticides, underscores the need for biocontrol strategies grounded in microbial chemistry and ecology. Within this context, we investigated the agricultural potential of *Aspergillus sulphureoviridis*, a chemically underexplored member of *Aspergillus* section Nidulantes (36). The strong antibacterial activity of its crude extract against both a Gram-negative phytopathogen (*D. solani*) and a Gram-positive model bacterium (*B. subtilis*) suggested a dual ecological function: direct antagonism via antibacterial metabolites and indirect modulation of bacterial defense responses. Our results support this hypothesis: *A. sulphureoviridis* produced potent antibacterial metabolites and, during co-culture, elicited defensive lipopeptide production in *B. subtilis*, pointing to a potential chemical interaction that warrants further evaluation in plant-associated systems.

By cultivating *A. sulphureoviridis* on different media to activate diverse biosynthetic pathways, we revealed a chemically rich secondary metabolome comprising bioactive ophiobolins, nidulanins, and emericellamides (Table 1). Although most of these compounds were not responsible for the observed antibacterial effects, their annotation demonstrated the fungus’s remarkable biosynthetic potential and narrowed the pool of bioactive candidates. Importantly, the integration of bioactivity-based molecular networking proved pivotal for prioritizing metabolites within this complex chemical space (10). This strategy, successfully employed in other natural product systems, enabled us to correlate MS features with bioactivity, avoiding the rediscovery of inactive metabolites and streamlining the identification of key antibacterial constituents (37). Through this approach, we identified F9775B (**5**) as a potent anti-phytobacterial metabolite active against *D. solani* (IC_₅₀_ = 59.3 µg/mL), a discovery of direct agricultural relevance. Originally reported from *Paecilomyces carneus* and *A. nidulans* as cathepsin K inhibitors, F9775B (**5**) is shown here to be a previously underappreciated antibacterial metabolites (34, 38). Moreover, this study expands the known chemical diversity of *Aspergillus* by reporting the first occurrence of cladobotrins from this genus, including two new analogs, cladobotrin VII (**2**) and VIII (**3**). Previously, these compounds were only known from *Cladobotryum* and *Fusarium* species (32, 39). Previous structure-activity relationship studies on related pyrone derivatives highlighted the importance of a C-5 formyl group for antifungal activity against *Ganoderma lucidum*, whereas analogs lacking this group (e.g., cladobotrin VI) were inactive (32). In our antibacterial assays, cladobotrin IV (**1**) and VII (**2**)—both lacking the C-5 formyl group—showed only weak inhibition of *B. subtilis* (≤50% at 100 µg/mL). In contrast, cladobotrin VIII (**3**), with its methyl formate substitution, exhibited moderate activity (IC_₅₀_ ≈ 95 µg/mL). The moderate activity of **3** compared to its analogs highlights the sensitivity of biological function to minor chemical modifications and underscores the potential for rational optimization of this scaffold.

In addition to these characterized metabolites, two further compounds with *m/z* 519.1286 (**7**) and *m/z* 521.1448 (**8**) were detected as novel, low-abundance features (Table 3). Although their complete structures could not be fully elucidated due to limited quantities, their molecular weights and MS/MS fragmentation patterns were confidently established. Molecular networking suggested that these two metabolites are structurally related to violaceol I (**6**) and may originate from a common biosynthetic pathway. These findings point to a possible expansion of the violaceol-related chemical space. Given their network proximity and bioactivity-linked clustering, these metabolites may possess strong antibacterial properties or act synergistically within the same metabolic network. Future studies will focus on their purification, structure elucidation, and evaluation of their bioactivity and ecological roles.

The ecological dimension of *A. sulphureoviridis* became evident from its interaction with *B. subtilis*. In natural environments, microbial communities engage in complex competitive and cooperative interactions that shape secondary metabolism. Co-cultivation is well known to awaken silent biosynthetic pathways or alter metabolite output through chemical communication. For instance, Wu et al. observed that co-culturing *B. amyloliquefaciens* with the fungus *Trichoderma* led to significantly higher levels of antibacterial compounds than monoculture (40–42). Similarly, our MALDI-MSI analysis revealed that *A. sulphureoviridis* acts not merely as an antagonist, but as a potent elicitor that reprograms *B. subtilis* metabolism (12).

The spatial distribution of the cyclic lipopeptides deployed against *A. sulphureoviridis* indicates the dynamic interplay between the bacterium and the fungus. The well-described *Bacillus* lipopeptides, surfactins and fengycins, were enriched during co-culture. Most intriguingly, we observed putative short-chain fengycin-like lipopeptides in the MSI data but only intracellularly within the *Bacillus* colony. It is understood that lipid chain length plays a key role in biological activity, with short chains losing activity entirely (43, 44). It remains unclear whether *Bacillus* selectively restricts the secretion of these smaller fengycin-like lipopeptides or whether they serve a distinct ecological function. One possible interpretation is that this intracellular retention reflects a resource-conserving response, in which *B. subtilis* preferentially deploys canonical surfactins and fengycins at the confrontation zone while retaining lower-activity or incompletely modified fengycin-like congeners within the colony. Alternatively, limiting the extracellular release of these membrane-active molecules may reduce unnecessary self-associated stress. We note that similar fengycin-related *m/z* values have been reported previously (45); however, those ions were observed as [M + H]^+^ species, whereas the ions detected in our study predominantly occur as [M + K]^+^ adducts. Given that our assignments are supported by consistent mass offsets across multiple adduct types, we consider it likely that the species observed here represent distinct congeners, rather than previously described variants.

Furthermore, the MALDI–MSI data demonstrate that F9775B (**5**) is spatially enriched at the fungal–bacterial interface. Under non-interacting conditions, F9775B is produced within the fungal mycelium, whereas extracellular localization is released at the confrontation zone. When considered alongside its *in vitro* antibacterial activity against *D. solani*, these findings are consistent with a role for F9775B in competitive interactions within mixed microbial communities.

**Fig 8:**
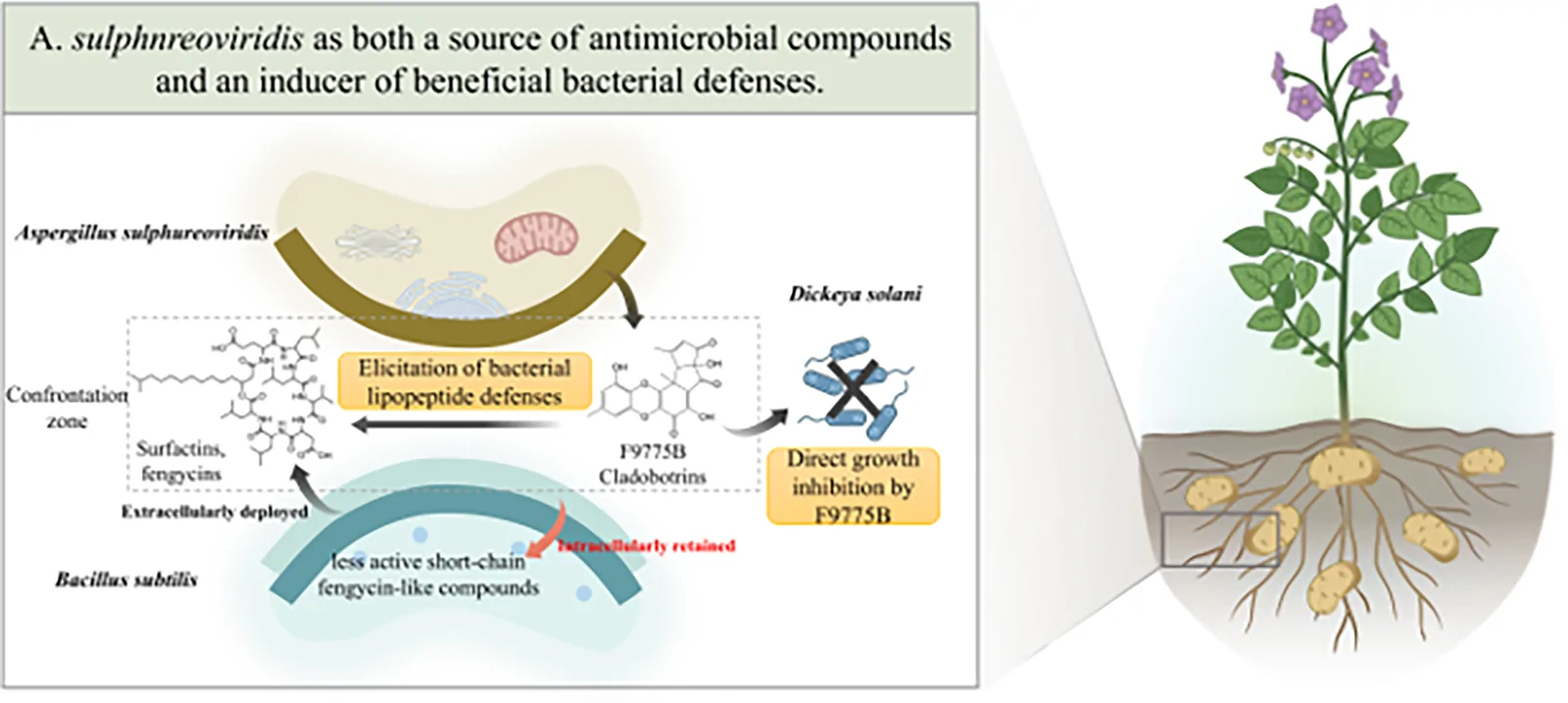
Proposed model of molecule-mediated interactions between *Aspergillus sulphureoviridis* and *Bacillus subtilis*. The schematic illustrates a dual-function biocontrol model in which *A. sulphureoviridis* produces antibacterial secondary metabolites, including the polyketide F9775B, that suppress the phytopathogen *Dickeya solani*. Simultaneously, fungal-derived chemical cues elicit lipopeptide-based defenses in *B. subtilis*, characterized by extracellular deployment of canonical surfactins and fengycins at the interaction interface, while shortened fengycin-like variants are retained intracellularly and exhibit reduced bioactivity.

In conclusion, this study establishes *A. sulphureoviridis* as a multifaceted microbial resource with dual agricultural relevance: a producer of direct-acting anti-phytopathogenic metabolites such as F9775B and an elicitor of systemic bacterial defenses (Fig. 8). These findings support further evaluation of *A. sulphureoviridis* as a potential component of microbe-based biocontrol strategies. Future investigations should focus on isolating and characterizing the newly induced bacterial lipopeptides, assessing synergistic metabolite combinations *in planta*, and exploring whether *A. sulphureoviridis* metabolites can also enhance plant growth. Such integrative efforts may pave the way toward microbial consortia that couple disease suppression with crop resilience, advancing sustainable agriculture through chemical ecology-informed biocontrol.

## Data Availability

The molecular networking job on GNPS can be found at https://gnps.ucsd.edu/ProteoSAFe/status.jsp?task=a46a690d7ae74a27857f432e0644a64f.

## SUPPLEMENTAL MATERIAL

Supplemental figures spectrum.00977-26-s0001.docx Figures S1 to S17.

ASM does not own the copyrights to Supplemental Material that may be linked to, or accessed through, an article. The authors have granted ASM a non-exclusive, world-wide license to publish the Supplemental Material files. Please contact the corresponding author directly for reuse.

## References

[1] Golanowska M, Łojkowska E. 2016. A review on *Dickeya solani*, a new pathogenic bacterium causing loss in potato yield in Europe. BioTechnologia 97:109–127. doi:10.5114/bta.2016.60781

[2] Babinska-Wensierska W, Motyka-Pomagruk A, Fondi M, Misztak AE, Mengoni A, Lojkowska E. 2024. Differences in the constituents of bacterial microbiota of soils collected from two fields of diverse potato blackleg and soft rot diseases incidences, a case study. Sci Rep 14:18802. doi:10.1038/s41598-024-69213-w 39138329 PMC11322387

[3] Chen AJ, Frisvad JC, Sun BD, Varga J, Kocsubé S, Dijksterhuis J, Kim DH, Hong SB, Houbraken J, Samson RA. 2016. Aspergillus section nidulantes (formerly Emericella): polyphasic taxonomy, chemistry and biology. Stud Mycol 84:1–118. doi: 10.1016/j.simyco.2016.10.001 28050053 10.1016/j.simyco.2016.10.001 PMC5198626

[4] Houbraken J, Kocsubé S, Visagie CM, Yilmaz N, Wang X-C, Meijer M, Kraak B, Hubka V, Bensch K, Samson RA, Frisvad JC. 2020. Classification of aspergillus, penicillium, talaromyces and related genera (Eurotiales): an overview of families, genera, subgenera, sections, series and species. Stud Mycol 95:5–169. doi:10.1016/j.simyco.2020.05.002 32855739 PMC7426331

[5] Theobald S, Vesth T, Nybo JL, Frisvad JC, Kjærbølling I, Mondo S, LaButti K, Haridas S, Riley R, Kuo AA, et al.. 2025. Comparative genomics of aspergillus nidulans and section nidulantes. Current Research in Microbial Sciences 8:100342. doi:10.1016/j.crmicr.2025.100342 39897699 PMC11787670

[6] Feng HM, Xiang HJ, Yun WS, Qi TY. 2027. Research progress on new alkaloids of the genus aspergillus from 2019 to 2024. Food Med Homol 6867:3006–4252. doi:10.26599/FMH.2027.9420151

[7] Henke MT, Kelleher NL. 2016. Modern mass spectrometry for synthetic biology and structure-based discovery of natural products. Nat Prod Rep 33:942–950. doi:10.1039/c6np00024j 27376415 PMC4981503

[8] Wang M, Carver JJ, Phelan VV, Sanchez LM, Garg N, Peng Y, Nguyen DD, Watrous J, Kapono CA, Luzzatto-Knaan T, et al.. 2016. Sharing and community curation of mass spectrometry data with global natural products social molecular networking. Nat Biotechnol 34:828–837. doi:10.1038/nbt.3597 27504778 PMC5321674

[9] Pluskal T, Castillo S, Villar-Briones A, Oresic M. 2010. MZmine 2: modular framework for processing, visualizing, and analyzing mass spectrometry-based molecular profile data. BMC Bioinformatics 11:395. doi:10.1186/1471-2105-11-395 20650010 PMC2918584

[10] Nothias L-F, Nothias-Esposito M, da Silva R, Wang M, Protsyuk I, Zhang Z, Sarvepalli A, Leyssen P, Touboul D, Costa J, Paolini J, Alexandrov T, Litaudon M, Dorrestein PC. 2018. Bioactivity-based molecular networking for the discovery of drug leads in natural product bioassay-guided fractionation. J Nat Prod 81:758–767. doi:10.1021/acs.jnatprod.7b00737 29498278

[11] Sun Y, Liu W-C, Shi X, Zheng H-Z, Zheng Z-H, Lu X-H, Xing Y, Ji K, Liu M, Dong Y-S. 2021. Inducing secondary metabolite production of *Aspergillus sydowii* through microbial co-culture with *Bacillus subtilis*. Microb Cell Fact 20:42. doi:10.1186/s12934-021-01527-0 33579268 PMC7881642

[12] Hoefler BC, Gorzelnik KV, Yang JY, Hendricks N, Dorrestein PC, Straight PD. 2012. Enzymatic resistance to the lipopeptide surfactin as identified through imaging mass spectrometry of bacterial competition. Proc Natl Acad Sci USA 109:13082–13087. doi:10.1073/pnas.1205586109 22826229 PMC3420176

[13] Vallet M, Vanbellingen QP, Fu T, Le Caer J-P, Della-Negra S, Touboul D, Duncan KR, Nay B, Brunelle A, Prado S. 2017. An integrative approach to decipher the chemical antagonism between the competing endophytes *Paraconiothyrium variabile* and *Bacillus subtilis*. J Nat Prod 80:2863–2873. doi:10.1021/acs.jnatprod.6b01185 29139291

[14] Wang X, Subko K, Kildgaard S, Frisvad JC, Larsen TO. 2021. Mass Spectrometry-based network analysis reveals new insights into the chemodiversity of 28 species in *Aspergillus* section *Flavi*. Front Fungal Biol 2:719420. doi:10.3389/ffunb.2021.719420 37744124 PMC10512371

[15] Kildgaard S, Mansson M, Dosen I, Klitgaard A, Frisvad JC, Larsen TO, Nielsen KF. 2014. Accurate dereplication of bioactive secondary metabolites from marine-derived fungi by UHPLC-DAD-QTOFMS and a MS/HRMS library. Mar Drugs 12:3681–3705. doi:10.3390/md12063681 24955556 PMC4071597

[16] Shannon P, Markiel A, Ozier O, Baliga NS, Wang JT, Ramage D, Amin N, Schwikowski B, Ideker T. 2003. Cytoscape: a software environment for integrated models. Genome Res 13(11):2498–2504. doi:10.1101/gr.1239303 14597658 PMC403769

[17] Guo Y, Ding L, Ghidinelli S, Gotfredsen CH, de la Cruz M, Mackenzie TA, Ramos MC, Sánchez P, Vicente F, Genilloud O, Coriani S, Larsen RW, Frisvad JC, Larsen TO. 2021. Taxonomy driven discovery of polyketides from *Aspergillus californicus*. J Nat Prod 84:979–985. doi:10.1021/acs.jnatprod.0c00866 33656895

[18] Audoin C, Bonhomme D, Ivanisevic J, de la Cruz M, Cautain B, Monteiro MC, Reyes F, Rios L, Perez T, Thomas OP. 2013. Balibalosides, an original family of glucosylated sesterterpenes produced by the mediterranean sponge *Oscarella balibaloi*. Mar Drugs 11:1477–1489. doi:10.3390/md11051477 23648552 PMC3707155

[19] Bladt TT, Dürr C, Knudsen PB, Kildgaard S, Frisvad JC, Gotfredsen CH, Seiffert M, Larsen TO. 2013. Bio-activity and dereplication-based discovery of ophiobolins and other fungal secondary metabolites targeting leukemia cells. Molecules 18:14629–14650. doi:10.3390/molecules181214629 24287995 PMC6290568

[20] Yang JO, Nakayama N, Toda K, Tebayashi S, Kim CS. 2014. Structural determination of elicitors in sogatella furcifera (Horváth) that induce japonica rice plant varieties (Oryza sativa L.) to produce an ovicidal substance against *S. furcifera* eggs. Biosci Biotechnol Biochem 78:937–942. doi:10.1080/09168451.2014.917266 25036116

[21] Yamazaki M, Maebayashi Y. 1982. Structure determination of violaceol-I and -II, new fungal metabolites from a strain of *Emericella violacea*. Chem Pharm Bull 30:514–518. doi:10.1248/cpb.30.514

[22] Nuankeaw K, Chaiyosang B, Suebrasri T, Kanokmedhakul S, Lumyong S, Boonlue S. 2020. First report of secondary metabolites, violaceol i and violaceol II produced by endophytic fungus, *Trichoderma polyalthiae* and their antimicrobial activity. Mycoscience 61:16–21. doi:10.1016/j.myc.2019.10.001

[23] Maiya S, Grundmann A, Li X, Li SM, Turner G. 2007. Identification of a hybrid PKS/NRPS required for pseurotin a biosynthesis in the human pathogen *Aspergillus fumigatus*. Chembiochem 8:1736–1743. doi:10.1002/cbic.200700202 17722120

[24] Mille-Lindblom C, von Wachenfeldt E, Tranvik LJ. 2004. Ergosterol as a measure of living fungal biomass: persistence in environmental samples after fungal death. J Microbiol Methods 59:253–262. doi:10.1016/j.mimet.2004.07.010 15369861

[25] Klitgaard A, Nielsen JB, Frandsen RJN, Andersen MR, Nielsen KF. 2015. Combining stable isotope labeling and molecular networking for biosynthetic pathway characterization. Anal Chem 87:6520–6526. doi:10.1021/acs.analchem.5b01934 26020678

[26] Liu S-Z, Tang X-X, He F-M, Jia J-X, Hu H, Xie B-Y, Li M-Y, Qiu Y-K. 2022. Two new compounds from a mangrove sediment-derived fungus *Penicillium polonicum* H175. Nat Prod Res 36:2370–2378. doi:10.1080/14786419.2020.1837811 33146025

[27] Arias SL, Mary VS, Otaiza SN, Wunderlin DA, Rubinstein HR, Theumer MG. 2016. Toxin distribution and sphingoid base imbalances in *Fusarium verticillioides*-infected and fumonisin B1-watered maize seedlings. Phytochemistry 125:54–64. doi:10.1016/j.phytochem.2016.02.006 26903312

[28] Andersen MR, Nielsen JB, Klitgaard A, Petersen LM, Zachariasen M, Hansen TJ, Blicher LH, Gotfredsen CH, Larsen TO, Nielsen KF, Mortensen UH. 2013. Accurate prediction of secondary metabolite gene clusters in filamentous fungi. Proc Natl Acad Sci USA 110:E99–107. doi:10.1073/pnas.1205532110 23248299 PMC3538241

[29] Yang SX, Wang HP, Gao JM, Zhang Q, Laatsch H, Kuang Y. 2012. Fusaroside, a unique glycolipid from *Fusarium sp*., an endophytic fungus isolated from *Melia azedarach*. Org Biomol Chem 10:819–824. doi:10.1039/c1ob06426f 22124543

[30] Szewczyk E, Chiang YM, Oakley CE, Davidson AD, Wang CCC, Oakley BR. 2008. Identification and characterization of the asperthecin gene cluster of *Aspergillus nidulans*. Appl Environ Microbiol 74:7607–7612. doi:10.1128/AEM.01743-08 18978088 PMC2607171

[31] Oh DC, Kauffman CA, Jensen PR, Fenical W. 2007. Induced production of emericellamides a and b from the marine-derived fungus *Emericella sp*. in competing co-culture. J Nat Prod 70:515–520. doi:10.1021/np060381f 17323993

[32] Tezuka Y, Huang Q, Kikuchi T, Nishi A, Tubaki K. 1994. Studies on the metabolites of mycoparasitic fungi. I. Metabolites of *Cladobotryum varium*. Chem Pharm Bull 42:2612–2617. doi:10.1248/cpb.42.2612

[33] Hanai K, Kuwae A, Takai T, Senda H, Kunimoto KK. 2001. A comparative vibrational and NMR study of cis-cinnamic acid polymorphs and trans-cinnamic acid. Spectrochim Acta A Mol Biomol Spectrosc 57:513–519. doi:10.1016/s1386-1425(00)00401-7 11300563

[34] Sanchez JF, Chiang Y-M, Szewczyk E, Davidson AD, Ahuja M, Elizabeth Oakley C, Woo Bok J, Keller N, Oakley BR, Wang CCC. 2010. Molecular genetic analysis of the orsellinic acid/F9775 gene cluster of *Aspergillus nidulans*. Mol Biosyst 6:587–593. doi:10.1039/b904541d 20174687 PMC2903553

[35] Sajitha KL, Dev SA, Maria Florence EJ. 2016. Identification and characterization of lipopeptides from *Bacillus subtilis* B1 against sapstain fungus of rubberwood through MALDI-TOF-MS and RT-PCR. Curr Microbiol 73:46–53. doi:10.1007/s00284-016-1025-9 27004481

[36] Alburae NA, Mohammed AE, Alorfi HS, JamanTurki A, Asfour HZ, Alarif WM, Abdel-Lateff A. 2020. Nidulantes of aspergillus (Formerly Emericella): a treasure trove of chemical diversity and biological activities. Metabolites 10:73. doi:10.3390/metabo10020073 32079311 PMC7073611

[37] Fan B, Dewapriya P, Li F, Blümel M, Tasdemir D. 2020. Pyrenosetins a-c, new decalinoylspirotetramic acid derivatives isolated by bioactivity-based molecular networking from the seaweed-derived fungus *Pyrenochaetopsis* sp. FVE-001. Mar Drugs 18:47. doi:10.3390/md18010047 31940767 PMC7024310

[38] Schroeckh V, Scherlach K, Nützmann H-W, Shelest E, Schmidt-Heck W, Schuemann J, Martin K, Hertweck C, Brakhage AA. 2009. Intimate bacterial-fungal interaction triggers biosynthesis of archetypal polyketides in *Aspergillus nidulans*. Proc Natl Acad Sci USA 106:14558–14563. doi:10.1073/pnas.0901870106 19666480 PMC2732885

[39] Shiono Y, Shibuya F, Koseki T Harizon Supratman U, Uesugi S, Kimura K-I. 2015. A new α-pyrone metabolite from a mangrove plant endophytic fungus, Fusarium sp. J Asian Nat Prod Res 17:403–408. doi:10.1080/10286020.2014.961919 25355135

[40] Kunst F, Ogasawara N, Moszer I, Albertini AM, Alloni G, Azevedo V, Bertero MG, Bessières P, Bolotin A, Borchert S, et al.. 1997. The complete genome sequence of the gram-positive bacterium *Bacillus subtilis*. Nature 390:249–256. doi:10.1038/36786 9384377

[41] Marmann A, Aly AH, Lin W, Wang B, Proksch P. 2014. Co-cultivation--a powerful emerging tool for enhancing the chemical diversity of microorganisms. Mar Drugs 12:1043–1065. doi:10.3390/md12021043 24549204 PMC3944530

[42] Wu Q, Ni M, Dou K, Tang J, Ren J, Yu C, Chen J. 2018. Co-culture of *Bacillus amyloliquefaciens* ACCC11060 and *Trichoderma asperellum* GDFS1009 enhanced pathogen-inhibition and amino acid yield. Microb Cell Fact 17:155. doi:10.1186/s12934-018-1004-x 30285749 PMC6171294

[43] Grifé-Ruiz M, Hierrezuelo-León J, de Vicente A, Pérez-García A, Romero D. 2025. Diversification of lipopeptide analogues drives versatility in biological activities. J Agric Food Chem 73:1403–1416. doi:10.1021/acs.jafc.4c11372 39760433 PMC11741111

[44] Aoki R, Kumagawa E, Kamata K, Ago H, Sakai N, Hasunuma T, Taoka N, Ohta Y, Kobayashi S. 2025. Engineering of acyl ligase domain in non-ribosomal peptide synthetases to change fatty acid moieties of lipopeptides. Commun Chem 8:17. doi:10.1038/s42004-024-01379-w 39838140 PMC11751314

[45] Lin L-Z, Zheng Q-W, Wei T, Zhang Z-Q, Zhao C-F, Zhong H, Xu Q-Y, Lin J-F, Guo L-Q. 2020. Isolation and characterization of fengycins produced by *Bacillus amyloliquefaciens* JFL21 and its broad-spectrum antimicrobial potential against multidrug-resistant foodborne pathogens. Front Microbiol 11:579621. doi:10.3389/fmicb.2020.579621 33391199 PMC7775374

