# Supplementary material for: Dual-function Aspergillus sulphureoviridis: antibacterial polyketides against Dickeya solani and elicitation of Bacillus subtilis lipopeptides
Source: Microbiol Spectr. 2026 Jul 20;14(9):e00977-26. doi: 10.1128/spectrum.00977-26 (PMC13532338; doi:10.1128/spectrum.00977-26)
Supplement: Supplementary file 1 [file spectrum.00977-26-s0001.docx]

**Supplementary Information**

**Dual-function Aspergillus sulphureoviridis: antibacterial polyketides against Dickeya solani and elicitation of Bacillus subtilis lipopeptides**

**Xinhui Wang,^1,2*^ Carlos N. Lozano-Andrade,^2^ Scott A. Jarmusch,^2^ Jens C. Frisvad,^2^ Ákos T. Kovács^2,#^ and Thomas O. Larsen^2*^**

^1^Collaborative Innovation Center for Modern Grain Circulation and Safety, and College of Food Science and Engineering, Nanjing University of Finance and Economics, Nanjing, China; ^2^DTU Bioengineering, Technical University of Denmark, Kgs. Lyngby, Denmark.

**^*^Correspondence:** Xinhui Wang, wangxinhui_hi@163.com; Thomas O. Larsen, [tol@bio.dtu.dk](mailto:tol@bio.dtu.dk)

^#^Current affiliation: Institute of Biology, Leiden University, Sylviusweg 72, 2333BE Leiden, The Netherlands

**Figure S1.** Agar diffusion bioassay of YES fractions from *Aspergillus sulphureoviridis* against *Dickeya solani*.

**Figure S2.** Agar diffusion bioassay of YES fractions from *Aspergillus sulphureoviridis* against *Bacillus subtilis*

**Figure S3.** Molecular Networking of the fractionated *A. sulphureoviridis* extract against *D. solani*

**Figure S4.** Molecular Networking of the fractionated *A. sulphureoviridis* extract against *B. subtilis*

**Figure S5.** ^1^H NMR (800 MHz) Spectrum of Cladobotrin IV (**1**) in CDCl_3_

**Figure S6.** ^13^C NMR (200 MHz) Spectrum of Cladobotrin IV (**1**) in CDCl_3_

**Figure S7.** HSQC Spectrum of Cladobotrin IV (**1**) in CDCl_3_

**Figure S8.** HMBC Spectrum of Cladobotrin IV (**1**) in CDCl_3_

**Figure S9.** ^1^H NMR (800 MHz) Spectrum of Cladobotrin VII (**2**) in CD_3_OD

**Figure S10.** HSQC Spectrum of Cladobotrin VII (**2**) in CD_3_OD

**Figure S11.** HMBC Spectrum of Cladobotrin VII (**2**) in CD_3_OD

**Figure S12.** ^1^H NMR (800 MHz) Spectrum of VIII (**3**) in CDCl_3_

**Figure S13.** ^13^C NMR (200 MHz) Spectrum of VIII (**3**) in CDCl_3_

**Figure S14.** HSQC Spectrum of VIII (**3**) in CDCl_3_

**Figure S15.** HMBC Spectrum of VIII (**3**) in CDCl_3_

**Figure S16.** Antagonism between *A. sulphureoviridis* and *B. subtilis* on CYA solid media

**Figure S17.** AntiSMASH analysis of the *Bacillus subtilis* 3610 genome

**Figure S1.** Agar diffusion bioassay of YES fractions from *Aspergillus sulphureoviridis* against *Dickeya solani*. The test concentration of each fraction was 5 mg/mL, and 80 µL aliquots (0.40 mg) were applied per well in triplicate assays. Methanol served as the negative control, and kanamycin was used as the positive control. Among the twelve YES fractions, fractions 5–8 exhibited the strongest antibacterial activity (>50% inhibition) against *D. solani*, whereas the remaining fractions showed weak or no inhibition.

**Figure S2.** Agar diffusion bioassay of YES fractions from *Aspergillus sulphureoviridis* against *Bacillus subtilis*. Each fraction was tested at a concentration of 5 mg/mL, and 80 µL aliquots (0.40 mg) were applied per well in triplicate assays. Kanamycin served as the positive control, while YES medium and methanol were used as negative controls. Among the twelve YES fractions, fractions 5–8 exhibited the strongest antibacterial activity (> 50% inhibition) against *B. subtilis*, whereas the remaining fractions showed weak or no inhibition.

**Figure S3.** Bioactivity-based molecular network of the fractionated *Aspergillus sulphureoviridis* extract against *Dickeya solani*.

**Figure S4.** Bioactivity-based molecular network of the fractionated *Aspergillus sulphureoviridis* extract against *Bacillus subtilis*.

**Figure S5.**^1^H NMR (800 MHz) Spectrum of Cladobotrin IV (**1**) in CDCl_3_

**Figure S6.** ^13^C NMR (200 MHz) Spectrum of Cladobotrin IV (**1**) in CDCl_3_

**Figure S7.** HSQC Spectrum of Cladobotrin IV (**1**) in CDCl_3_

**Figure S8.** HMBC Spectrum of Cladobotrin IV (**1**) in CDCl_3_

**Figure S9.** ^1^H NMR (800 MHz) Spectrum of Cladobotrin VII (**2**) in CD_3_OD

**Figure S10.** HSQC Spectrum of Cladobotrin VII (**2**) in CD_3_OD

**Figure S11.** HMBC Spectrum of Cladobotrin VII (**2**) in CD_3_OD

**Figure S12.** ^1^H NMR (800 MHz) Spectrum of cladobotrin VIII (**3**) in CDCl_3_

**Figure S13.** ^13^C NMR (200 MHz) Spectrum of cladobotrin VIII (**3**) in CDCl_3_

**Figure S14.** HSQC Spectrum of cladobotrin VIII (**3**) in CDCl_3_

**Figure S15.** HMBC Spectrum of cladobotrin VIII (**3**) in CDCl_3_

**Figure S16.** Antagonism between *A. sulphureoviridis* and *B. subtilis* on CYA solid media

**Figure S17.** AntiSMASH analysis of the *Bacillus subtilis* 3610 genome. The analysis identified 15 putative secondary metabolite biosynthetic regions under relaxed stringency. Among these, two NRPS clusters correspond to known lipopeptide pathways: Region 1.2 encodes the surfactin biosynthetic cluster and Region 1.5 encodes the fengycin cluster. No additional NRPS regions showed high-confidence matches to characterized lipopeptide BGCs in the MIBiG database.
